# Extragenic Accumulation of RNA Polymerase II Enhances Transcription by RNA Polymerase III

**DOI:** 10.1371/journal.pgen.0030212

**Published:** 2007-11-23

**Authors:** Imke Listerman, Anita S Bledau, Inna Grishina, Karla M Neugebauer

**Affiliations:** Max-Planck Institute of Molecular Cell Biology and Genetics, Dresden, Germany; Yale University, United States of America

## Abstract

Recent genomic data indicate that RNA polymerase II (Pol II) function extends beyond conventional transcription of primarily protein-coding genes. Among the five snRNAs required for pre-mRNA splicing, only the U6 snRNA is synthesized by RNA polymerase III (Pol III). Here we address the question of how Pol II coordinates the expression of spliceosome components, including U6. We used chromatin immunoprecipitation (ChIP) and high-resolution mapping by PCR to localize both Pol II and Pol III to snRNA gene regions. We report the surprising finding that Pol II is highly concentrated ∼300 bp upstream of all five active human U6 genes in vivo. The U6 snRNA, an essential component of the spliceosome, is synthesized by Pol III, whereas all other spliceosomal snRNAs are Pol II transcripts. Accordingly, U6 transcripts were terminated in a Pol III-specific manner, and Pol III localized to the transcribed gene regions. However, synthesis of both U6 and U2 snRNAs was α-amanitin-sensitive, indicating a requirement for Pol II activity in the expression of both snRNAs. Moreover, both Pol II and histone tail acetylation marks were lost from U6 promoters upon α-amanitin treatment. The results indicate that Pol II is concentrated at specific genomic regions from which it can regulate Pol III activity by a general mechanism. Consequently, Pol II coordinates expression of all RNA and protein components of the spliceosome.

## Author Summary

During transcription, RNA polymerases synthesize an RNA copy of a given gene. Human genes are transcribed by either RNA polymerase I, II, or III. Here, we focus on transcription of the U6 gene that encodes a small nuclear RNA (snRNA), a non-coding RNA with unique activities in gene expression. The U6 snRNA is transcribed by RNA polymerase III (Pol III); here we report the surprising finding that RNA polymerase II (Pol II) is important for efficient expression of the U6 snRNA. Interestingly, high concentrations of Pol II have been recently observed on genomic regions that are considered outside of transcribed genes. We localized Pol II to a region upstream of the U6 snRNA gene promoters in living cells. Inhibition of Pol II activity decreased U6 snRNA synthesis and was accompanied by a decrease in Pol II accumulation as well as transcription-activating histone modifications, while Pol III remained bound at U6 genes. Thus, Pol II may promote U6 snRNA transcription by facilitating open chromatin formation. Our results provide insight into the extragenic function of Pol II, which can coordinate the expression of all components of the RNA splicing machinery, including U6 snRNA.

## Introduction

The spliceosome is a multicomponent complex of five small nuclear RNAs (snRNAs) and ∼250 proteins that assemble on pre-mRNA to catalyze intron removal [1]. U6 snRNA is the shortest and least variable of the spliceosomal snRNAs, reflecting its central role in the splicing process [2]. It is a very dynamic molecule undergoing multiple conformational changes during assembly and splicing. Together with Lsm proteins and Prp24, U6 snRNA forms the U6 small nuclear ribonucleoparticle (snRNP) and is also found base paired with U4 snRNA in the U4/U6 di-snRNP and in the U4/U6·U5 tri-snRNP [3,4]. U6 snRNA is an exceptional member of the spliceosomal snRNAs. While U1, U2, U4, and U5 snRNAs are synthesized by RNA polymerase II (Pol II) and contain a 2,2,7-trimethylguanosine (TMG) cap at their 5′ ends, U6 snRNA is synthesized by RNA polymerase III (Pol III) and contains a γ-monomethyl phosphate cap. Furthermore, the U6 snRNA 3′ end is posttranscriptionally uridylated and blocked with a 2′,3′-cyclic phosphate [5–8]. These features are shared among some Pol III transcribed snRNAs, such as 7SK and H1. Direct evidence that the U6 snRNA gene is transcribed by Pol III relies upon its α-amanitin insensitivity in vitro. U6 snRNA gene transcription in S100 extracts and isolated nuclei was not sensitive to low α-amanitin concentrations that selectively inhibit Pol II but not Pol III. Furthermore, a cloned human U6 snRNA gene could be transcribed in a HeLa S100 extract lacking Pol II activity [5,8]. Pol III transcription of U6 snRNA genes was reported to occur in Xenopus tropicalis oocytes, Saccharomyces cerevisiae and Schizosaccharomyces pombe [9–12]. Other evidence for Pol III-directed U6 snRNA gene transcription is the fact that the gene ends with a stretch of five thymidine residues, a common Pol III transcription termination site [13,14].

The snRNA genes are highly interesting because they have very similar promoters yet are transcribed by two different polymerases [15]. All vertebrate snRNA gene promoters contain a distal sequence element (DSE) ∼220 bp upstream of the initiation site that functions as an enhancer and a proximal sequence element (PSE) −60 bp that is a core promoter element. The PSE and DSE recruit the same set of transcription factors to the different snRNA genes. The snRNA activator protein complex (SNAPc) binds to the PSE and nucleates the assembly of the transcription initiation complex, whereas the DSE contains OCT and SPH elements that serve as binding sites for Oct-1 and Staf transcription factors. A special feature of the U6 snRNA promoter is the presence of a TATA box at −25 bp, which binds TBP in the TFIIIB complex and leads to recruitment of Pol III [16]. The U6 promoter can drive transcription by Pol II under certain circumstances, such as mutation in the U6 snRNA gene TATA box; conversely, insertion of a TATA box into the U2 snRNA promoter converts this Pol II promoter into a template for Pol III [9,17–19]. Thus, the TATA box, present in all identified human U6 genes, is responsible for the Pol III specificity of the U6 snRNA promoters [20,21].

The expectation generated by these mechanistic studies is that Pol III—not Pol II—will be recruited to active U6 genes. Yet, here we show that the Pol II large subunit is highly concentrated close to the DSE ∼300 bp upstream of the transcription start sites in all five known active U6 loci in vivo. Further experiments indicate that the Pol II present at U6 promoters plays a positive role in the synthesis of U6 snRNA by Pol III, without affecting Pol III recruitment.

## Results

### Pol II and Pol III Accumulate in Distinct Peaks at U6 snRNA Gene Promoters In Vivo

**Figure 1:**
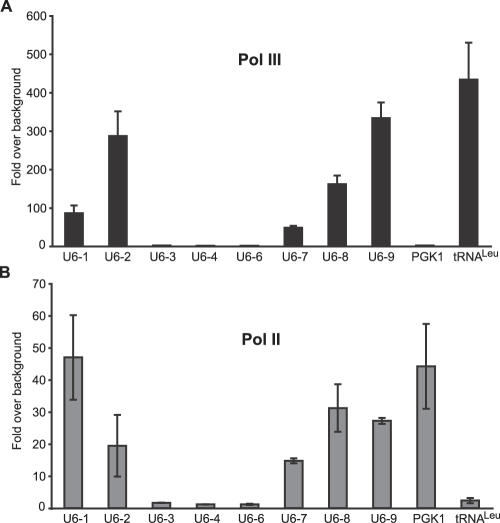
Both Pol II and Pol III Accumulate at Active *U6 snRNA* Gene Promoters Sheared chromatin from pemHeLa cells that had been crosslinked with formaldehyde was immunoprecipitated with (A) anti-Pol III (1900) or (B) anti-Pol II antibodies (8WG16). DNA was purified following reversal of crosslinks. qPCR primers specific for regions ∼ 300 bp upstream of active and inactive *U6* genes (*U6–1*, *U6–2*, *U6–7*, *U6–8*, *U6–9* and *U6–3*, *U6–4*, *U6–6*, respectively), *PGK1*, and *tRNA^Leu^* genes (Pol II and Pol III controls, respectively). All values are relative to nonimmune IgG and normalized to an intergenic control region. Error bars represent the SEM of at least three independent experiments.

Human U6 snRNA is expressed from several genes, five of which have been identified so far. Four apparently inactive U6 genes are also present in the genome [22]. Using chromatin immunoprecipitation (ChIP), we detected robust accumulation of Pol III at promoters of the active U6 snRNA genes (*U6–1*, *U6–2*, *U6–7*, *U6–8* and *U6–9*) but not at the promoters of the inactive genes (*U6–3*, *U6–4* and *U6–6*) in HeLa cells (Figure 1A). As expected, Pol III levels on the active *U6* and *tRNA^Leu^* genes were comparable. Interestingly, the *U6–7* promoter, which exhibits the weakest Pol III occupancy, contains a suboptimal PSE and was shown to be transcriptionally less active in vivo compared to other *U6 snRNA* genes [22]. Thus, the extent of Pol III accumulation on the nine *U6* genes correlates with their transcriptional activity.

Unexpectedly, when we performed ChIP with a Pol II-specific antibody, we detected robust Pol II accumulation at all active *U6* gene promoters but not on the inactive ones (Figure 1B). The extent of Pol II occupancy on the various *U6* promoters was comparable to the highly active *phosphoglycerate kinase* (*PGK1*) gene promoter. To examine the genomic regions surrounding the *U6* genes, we consulted the UCSC human genome browser [23,24]. Start sites of protein coding genes mapped close to *U6–9* and *U6–2* and were shown to be transcriptionally active (Figure S1 and unpublished data); consequently, the Pol II signals on the *U6–2* and *U6–9* promoters might be partly derived from these Pol II genes. In contrast, the only neighboring gene of *U6–1* (*BC033162*, ∼1 kb downstream of the *U6–1* transcription start site) was transcriptionally inactive (Figure S2A). Furthermore, no transcripts derived from sequences 800 bp upstream or downstream of *U6–1* were detected by Northern blotting with probes covering the + and − strands (Figure S2B). No genes were annotated near the active *U6–7* and *U6–8* genes, which lie 1 kb apart from each other on opposite strands of chromosome 14 (Figure S1). This indicates that Pol II accumulation on *U6–1*, *U6–7* and *U6–8 snRNA* gene promoters is not due to the presence of neighboring Pol II promoters. Therefore, all subsequent experiments focus on these three *U6* genes. The high levels of Pol II occupancy on *U6* promoters and the observation that both polymerases are present only on active *U6* gene promoters suggest that both polymerases may have a function in *U6* gene expression and ultimately spliceosome regulation.

**Figure 2:**
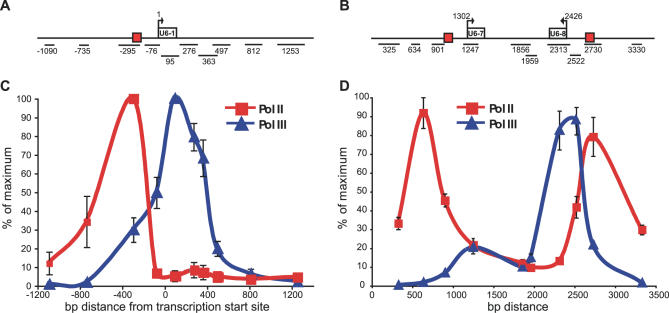
Pol II and Pol III Accumulate in Two Distinct Peaks at the *U6–1*, *U6–7*, and *U6–8* Promoters (A, B) Diagrams of the *U6–1* and *U6–7/U6–8* gene regions, with black lines specifying the PCR amplicons identified by the central nucleotide. Boxes represent the position of the DSE. (C, D) Summary plots of ChIP experiments with Pol II and Pol III distributions on *U6–1* gene region (C) and *U6–7* and *U6–8* (D). Crosslinked pemHeLa extracts were used for ChIP with primers amplifying fragments distributed 1 kb upstream and downstream of each gene. Antibodies specific for Pol II (4H8) and Pol III were used. X-axis values in (C) are relative to the *U6–1* transcription start site at position +1, whereas transcription start sites in (D) are at +1,302 (U6–7) and +2,426 (U6–8), relative to an arbitrary point upstream of *U6–7*. Data points are placed according to the center positions of the PCR products along the region. The peak value for the region (either *U6–1* region or *U6–7/U6–8* region) was determined for each biological replicate and set to 100%; displayed values are averaged among at least three biological replicates. Error bars represent the SEM.

To further investigate this unforeseen behavior of Pol II, the accumulation pattern of the two polymerases was mapped in greater detail on the *U6–1*, *U6–7*, and *U6–8* gene regions by ChIP. Strikingly, Pol II and Pol III accumulate in two distinct peaks on each of these three *U6* genes. Pol II is highly enriched 300–700 bp upstream of the transcription start site, whereas Pol III is most highly enriched over the U6 snRNA transcribed region (Figure 2A–2D and Figure S3). For Pol II ChIPs, we made use of two different, well-characterized antibodies that recognize either total Pol II (4H8, Figure S3 C, D) or the hypophosphorylated heptad repeat of the Pol II CTD (8WG16, Figure S3 E, F); both antibodies revealed Pol II accumulation in the same pattern on the *U6* gene promoter. Interestingly, Pol II occupies the region around the DSE that functions as an enhancer for snRNA transcription [25]. The sharp separation of Pol II and Pol III crosslinking signals indicates that Pol II and Pol III bind to distinct sequences in the *U6* promoter regions.

**Figure 3:**
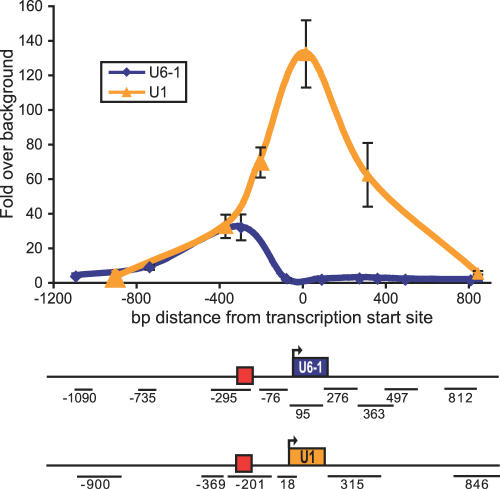
Comparison of Pol II Accumulation Pattern on *U6–1* and *U1* snRNA Genes ChIP with Pol II mAb 4H8 was performed with pemHeLa crosslinked extracts and the purified DNA subjected to qPCR with primers along the *U6–1* and *U1 snRNA* gene regions as shown (Ensembl ID ENSG00000194297 on chromosome 1). Data points represent the center point of the amplified region and Pol II fold accumulation over background and intergenic region, as in Figure 1. Diagrams below the plot indicate the features of both genes, which have been aligned at their respective transcription start sites (arrows). Error bars represent the SEM of at least three independent experiments.

Comparison of the Pol II distribution at the *U1* and *U6 snRNA* genes suggests that Pol III transcribes the *U6* genes examined. Both *U1* and *U6* promoters contain the DSE at approximately −220 bp and the PSE at −60 bp. However, the *U1* promoter lacks a TATA box and is transcribed by Pol II. As shown in Figure 3, Pol II is massively detectable on the *U1 snRNA* transcribed region, thus resembling the accumulation pattern of Pol III on *U6* transcribed regions. Note that the Pol II peak at the *U1* gene promoter is very broad and encompasses the distal region; thus, Pol II is enriched approximately 30-fold above background near the DSEs of both *U1* and *U6* genes, suggesting that Pol II transcription complexes upstream of the transcription start site may play a role in the expression of all snRNA genes.

Phosphorylation of the Pol II C-terminal domain (CTD) is associated with transcriptional initiation and elongation activity [26]. Phosphorylated Serine 5 (Ser5) residues associated with initiation are most highly detectable in promoter-proximal regions [27]. A specific antibody (H14) for this CTD modification has been previously generated and characterized [28,29]. ChIP with H14 revealed that Pol II CTD on the *U6–1* promoter is phosphorylated, indicating that an active Pol II transcription complex may form upstream of the natural *U6* transcription start site (Figure S4).

### Pol II Activity Is Required for Proper U6 snRNA Expression In Vivo

**Figure 4:**
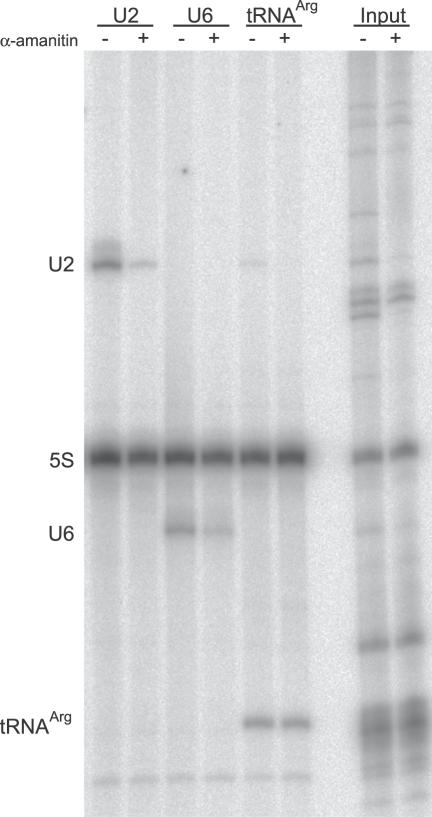
α-Amanitin Reduces Endogenous U6 snRNA Expression HeLa cells were transfected with a *tRNA^Arg^* maxigene and 16 h later treated with 10 μg/ml α-amanitin or left untreated. After 3 h, 250 μCi [^32^P] orthophosphate was added to the medium for 6 h. Total RNA was collected and 5S rRNA along with tRNA^Arg^, U2 and U6 snRNAs were hybrid selected with biotinylated complementary oligos. Hybrid-selected RNA was separated on a denaturating polyacrylamide gel and exposed to PhosphorImager plates. The 5S rRNA bands served as a loading control. Three biological replicates were analyzed and a representative gel is shown.

To investigate whether transcriptionally active Pol II plays a functional role in U6 snRNA biogenesis in vivo, we made use of the drug α-amanitin that specifically inhibits Pol II at low concentrations [30]. Structural and biochemical studies indicate that α-amanitin inhibits Pol II translocation along the DNA [31,32]. As the U6 snRNA is extremely stable with a half-life of at least 24 h [33], metabolic labeling was used to detect U6 snRNAs synthesized in the presence or absence of α-amanitin. Hybrid selection of endogenous U6 and U2 snRNA, 5S rRNA and plasmid-derived tRNA^Arg^ revealed distinct effects of α-amanitin on their expression. Figure 4 shows that the Pol III-driven genes 5S and tRNA were relatively unaffected by α-amanitin treatment, whereas both U2 and U6 snRNA levels were strongly reduced. Band intensities were normalized to 5S rRNA that served as an internal loading control. Quantification of three independent experiments showed that tRNA levels remained virtually constant after α-amanitin treatment (the mean ± standard error of the mean (SEM) = 84% ± 18% of the RNA remaining). In contrast, only 10% ± 2% of U2 snRNA and 48% ± 3% of U6 snRNA remained compared to untreated cells. Unpublished work from our lab showed that labeled U6 snRNA assembles with U6 and U4/U6 snRNP-specific proteins under conditions of α-amanitin treatment (data not shown), suggesting that U6 levels were not diminished due to a lack of sufficient snRNP proteins. This surprising result indicates that Pol II activity is required for full expression levels of endogenous U6 snRNA in vivo.

**Figure 5:**
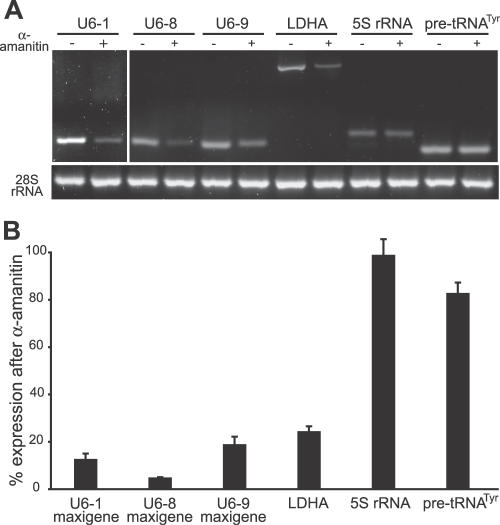
Low α-Amanitin Concentrations Inhibit U6 snRNA Maxigene Expression HeLa cells were transiently transfected with 1 μg *U6–1*, *U6–8*, and *U6–9* maxigenes carrying a 9 bp insertion and treated simultaneously with 50 nM α-amanitin oleate for 20 h or left untreated. Expression of *U6* maxigenes, LDHA, 28S rRNA, 5S rRNA and pre-tRNA^Tyr^ was measured by gene-specific reverse transcription, followed by conventional PCR and agarose gel electrophoresis (A) or qPCR (B). qPCR values were normalized to 28S rRNA and expression levels are expressed relative to untreated controls. Error bars represent the SEM. The data represents the average of at least three independent experiments.

Although nine *U6* genes have been identified in the human genome and their promoters studied [22], it is unclear how many *U6* genes might be present and contribute to the U6 species measured in the above metabolic labeling experiment. In order to specifically determine the action of α-amanitin-mediated Pol II inhibition on individual U6 snRNAs in their natural promoter context, we made use of plasmids harboring *U6* maxigenes with a 9 bp insert at the 3′ end of the transcribed region and the genomic 5′ and 3′ flanking regions including all regulatory sequences [22]. The 9 bp insert serves as a primer-binding site for reverse transcription and for subsequent quantitative PCR (qPCR) for exclusive detection of the plasmid-derived U6 snRNA. After α-amanitin treatment, the expression of *U6–9*, *U6–1* and *U6–8* maxigenes was severely decreased to 18%, 12% and 4% of controls, respectively (Figure 5). The endogenous Pol III transcripts, 5S rRNA and pre-tRNA^Tyr^_,_ were largely unaffected; as an additional control, transfection and expression of tRNA^Arg^ from a plasmid was also unaffected by α-amanitin (see Figure 4). Note that the failure of a *U6–4* maxigene to be transcribed [22] demonstrates that α-amanitin-sensitive transcription is specifically driven by the U6 promoters and not a background activity of the plasmid backbone. The drastic down-regulation of the *U6* maxigenes driven by their endogenous promoters clearly indicates that Pol II is required for expression of these genes.

### Role of Pol II in Pol III Transcription of U6 Genes

**Figure 6:**
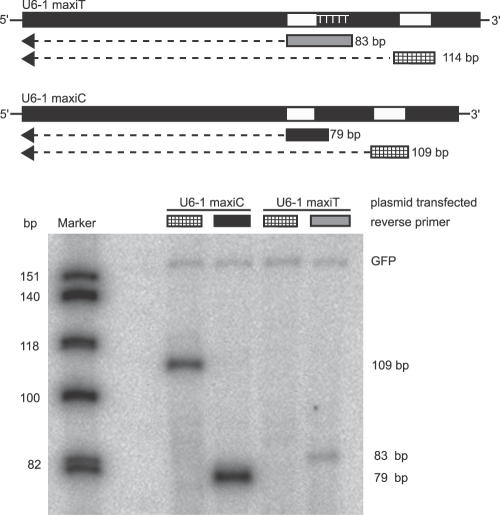
Pol III Transcribes U6–1 Maxigene Primer extension analysis of RNA from HeLa cells cotransfected with *GFP* expression plasmid and *U6–1* maxigene. (A) Design of the two *U6–1* maxigenes with insertions at +66 and +87 bp (white boxes) to allow for maxigene-specific reverse transcription. Construct “U6–1 maxiT” harbors five thymidine residues directly downstream of the linker insertion; “U6–1 MaxiC” harbors the same primer binding site but lacks the T's. The cross-hatched box represents the reverse primer specific for the downstream insertion, yielding extension products of either 114 bp (U6–1 maxiT) or 109 bp (U6–1 maxiC). Grey filled box and black filled box represent reverse primers specific for upstream insertion with or without T residues, respectively, leading to extension products of 83 bp (U6–1 maxiT) or 79 bp (U6–1 maxiC). Primer extension of mRNA derived from cotransfected GFP plasmid yields a 158 bp product. Primer extension products were separated on a denaturing polyacrylamide gel and exposed on a PhosphorImager. (B) A representative gel is shown.

One possible explanation for the above results is that Pol II might actually transcribe *U6 snRNA* genes. Previous work has shown that Pol II can transcribe from *U6 snRNA* promoters in human nuclear extracts and *Xenopus* oocytes [9,17,18]. Furthermore, human *U6* promoters used in short hairpin constructs can drive expression of luciferase and green fluorescent protein (GFP) [19]. In order to identify the polymerase responsible for actual U6 snRNA transcription, we designed *U6* maxigenes that harbor an additional insert midway through the transcribed region; this was used for specific primer extension analysis and was followed by a Pol III termination signal of five thymidine residues (see Figure 6). If Pol II transcribes the *U6* maxigene, transcription should not terminate at the early Pol III termination signal. If Pol III transcribes the maxigene, termination is expected to occur at the introduced termination site [13,14]. As shown in Figure 6, the short U6 snRNA transcript, but not the full-length transcript, was detected, strongly indicating that the *U6* maxigene is transcribed by Pol III. Note that the short, prematurely terminated U6 transcript was less abundant than the transcript generated from the control maxigene. Most likely this is because U6 snRNA stability requires 3′ end binding sites for processing factors and snRNP components (Lsm proteins) that are downstream of the inserted termination site [3,4]. Because Pol II activity was required for maxigene transcription (see Figure 5), it appears that Pol II controls *U6* gene transcription by Pol III.

**Figure 7:**
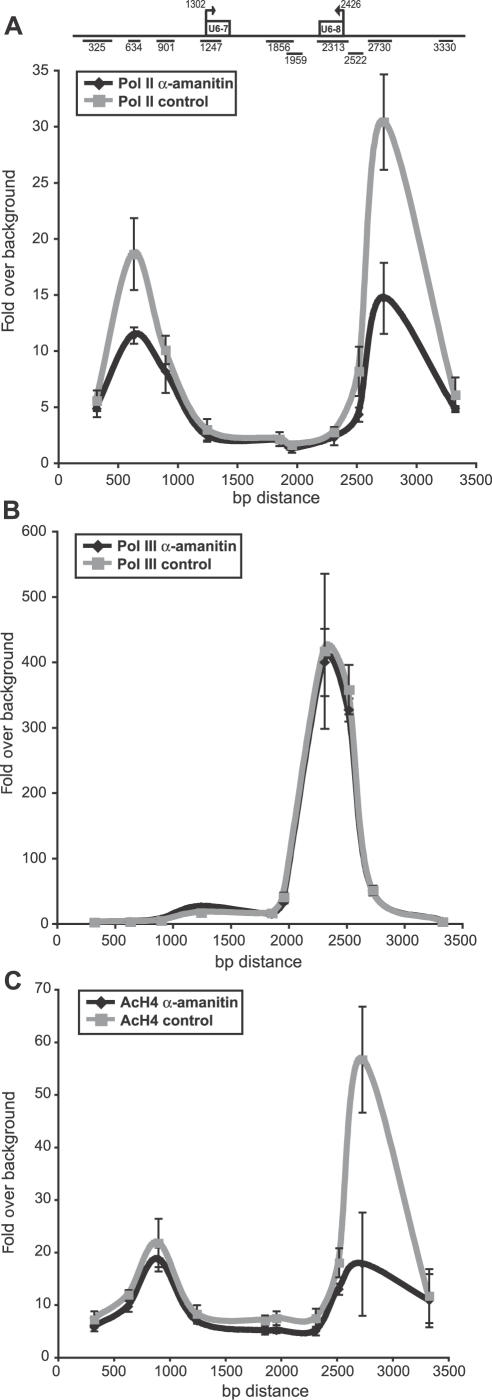
Pol II and Histone Acetylation Levels at U6 Genes Are Reduced by α-Amanitin Extracts from crosslinked Hela cells, either treated for 9 h with 10 μg/ml α-amanitin or left untreated, were subjected to ChIP with (A) Pol II, (B) Pol III, or (C) acetylated histone H4 antibodies. Purified DNA was subjected to qPCR with primers along *U6–7* and *U6–8* gene regions. All values are relative to background and normalized to an intergenic control region. Error bars represent the SEM. The data represents the average of three to five independent experiments.

To gain insight into how Pol II might influence Pol III-mediated *U6* gene expression, the effect of α-amanitin treatment on Pol II and Pol III distributions was examined. We performed ChIP on cells treated with α-amanitin as described in Figure 4. Pol III distribution on *U6–7* and *U6–8* was nearly unchanged (Figure 7B), whereas Pol II occupancy at the promoter was decreased to 50% after α-amanitin treatment (Figure 7A). These data indicate that Pol II is not required for the steady-state accumulation of Pol III at the *U6* promoter. Interestingly, a ∼50% reduction of U6 snRNA synthesis is observed under the same α-amanitin treatment conditions (see Figure 4), strongly arguing for a functional link between Pol II occupancy and U6 snRNA expression levels. The observations that Pol III occupancy does not change upon loss of Pol II and that Pol II is not involved in the actual transcription of U6 snRNA suggest that Pol II has a role in the regulation of Pol III activity.

Transcriptionally active Pol II genes are characterized by specific histone modifications, most prominently acetylation [26,34]. It is not well understood how chromatin at Pol III genes is modified; however, it was shown that histone H4 is acetylated at active *U6* genes [22]. Because Pol II may target histone modifying enzymes to chromatin [26,35,36], we tested whether histone H4 acetylation was affected by α-amanitin treatment. In the absence of α-amanitin, histone H4 acetylation was highest in the *U6–7* and *U6–8* promoter regions and less pronounced at the *U6* transcribed region, although still above background. Interestingly, after α-amanitin treatment, histone H4 acetylation in the promoter region of *U6–8* was dramatically reduced (Figure 7C). At the *PGK1* promoter, α-amanitin also decreased Pol II levels, while Pol III levels at the tRNA^Leu^ promoter were unaffected (data not shown). Thus, at this U6 promoter, α-amanitin treatment led to a concomitant loss of Pol II and histone modifications associated with transcriptional activity and chromatin open state. These data suggest that Pol II-mediated histone modifications may positively regulate Pol III transcription of *U6 snRNA* genes.

## Discussion

Genome-wide studies in yeast, mouse, and human cells have demonstrated that RNA polymerase II is not only associated with active or even inactive but well-annotated protein-coding genes [37,38]. Instead, many Pol II transcripts derive from intergenic regions [39], implying that Pol II is distributed throughout the genome at unexpected sites. Indeed, Pol II is enriched at many untranscribed genes in budding yeast [40] and quite dramatically at promoters of inactive genes in human ES cells [41]. In contrast, recruitment of the Pol III transcriptional machinery seems confined to known Pol III-driven genes and to reflect transcriptional activity, at least in yeast [42]. The present study documents the unanticipated location and function of RNA Pol II at Pol III transcription units, providing insights into the potential activities of Pol II elsewhere in the genome.

Surprisingly little is known about Pol III structure, function, or regulation [43–45]. The realization that Pol III activity can be regulated by trans-acting factors (e.g., c-myc, p53, Rb, Maf1) indicates that regulation of Pol III activity may be more complex than anticipated and that Pol II and Pol III regulatory mechanisms may be shared [46–50]. Another transcription regulatory mechanism that may be general to both polymerases is histone tail modification. Indeed, histone acetylation was found to dramatically increase transcription by Pol III in vitro [51]. Based on the evidence summarized below, we speculate that Pol II recruitment upstream of the *U6* transcription start site leads to the opening or remodeling of chromatin, thus facilitating Pol III initiation or elongation.

First, our data are consistent with the conclusion that Pol III transcribes the *U6 snRNA* genes. Both Pol II and Pol III are recruited to all active *U6* genes; however, Pol II is present on upstream promoter elements and not on transcribed regions. In contrast, Pol III is concentrated on the coding regions, strongly suggesting that Pol III is engaged in *U6* transcription. Moreover, the introduction of an early Pol III termination site into a *U6* maxigene led to premature termination of the transcript at the expected site, and longer transcripts indicative of Pol II mediated elongation past the introduced Pol III termination site were not detected. We were also unable to detect capped or longer forms of U6 snRNA that might have been transcribed by Pol II initiating from an alternative upstream promoter (IL&KN, unpublished data). Therefore, all of the available evidence indicates that U6 snRNA is synthesized by Pol III.

Second, we gathered compelling evidence that Pol II accumulation, and possibly activity, is essential for proper U6 snRNA expression. As detected by ChIP, Pol II large subunit is as abundant on the promoters of active *U6* genes as it is on highly expressed bona fide Pol II-driven genes. Pol II does not accumulate on inactive *U6* genes, linking transcriptional activity and Pol II occupancy. The transcription inhibitor α-amanitin binds to Pol II with high affinity and blocks its translocation on the DNA template [31,32]. We showed that individual, plasmid-borne *U6 snRNA* genes are highly α-amanitin sensitive, indicating that Pol II activity is required for *U6* gene expression. Importantly, α-amanitin strongly reduced endogenous U2 and U6 snRNA synthesis whereas 5S rRNA and tRNA levels remained constant under the same conditions. The latter observation makes it unlikely that decreased expression of specific transcription factors upon α-amanitin treatment accounts for the observed effects on *U6* transcription. The fact that Ser5 phosphorylation of the Pol II CTD was detected in the *U6–1* promoter region underscores the suggestion that Pol II may be transcriptionally active upstream of the *U6* transcriptional start site (see Figure S4). If so, Pol II likely synthesizes either very short and/or very unstable RNAs from this upstream position, because we were unable to detect transcripts derived from either strand (see Figure S2B).

Finally, α-amanitin treatment led to the loss of Pol II as well as histone H4 acetylation levels at the *U6–8* promoter. Histone acetylation is thought to maintain the unfolded structure of transcriptionally active chromatin [52]. Interestingly, at least two bona fide histone acetyltransferases associate with elongating Pol II [35,36], implying that Pol II can distribute acetylation marks along transcribed chromatin [53]. We speculate that Pol II recruitment to *U6* genes may direct activating histone modifications to *U6* promoters and thereby promote Pol III activity at *U6* loci. This hypothesis might explain the discrepancy between our in vivo data and the α-amanitin insensitivity of *U6* transcription from plasmids in vitro. In vivo, access to upstream promoter regions might be constrained by nucleosomal packaging, and the concomitant action of Pol II and histone acetyltransferases could facilitate the opening of chromatin and binding of factors relevant for Pol III initiation. Indeed, the presence of positioned nucleosome between the DSE and PSE may facilitate regulation of Pol III by Pol II via histone tail modification [54,55].

We considered the possibility that Pol II is required co- or post-transcriptionally for U6 snRNA expression. Pol II could be responsible for recruitment of U6 snRNA processing factors, such as U6 capping enzymes, La protein, 3′ nuclease, cyclic phosphatase, or uridyl transferase [6,7,56–59]. Recruitment of these factors, though so far not linked to Pol II in any way, could theoretically promote U6 snRNA processing and/or stability. However, metabolically labeled U6 snRNA does assemble with U6 and U4/U6 snRNP specific proteins under conditions of α-amanitin treatment (I.L. and K.M.N., unpublished results), suggesting that U6 snRNP proteins are not limiting. Consistent with this, U6 snRNA stability is not compromised by translational inhibitors [60]. Finally, U6 snRNP stability is not likely dependent on U4 snRNA expression, because the ratio of U4 and U6 snRNAs varies widely among cell types [61–63]. Taken together, we have no reason to suspect that U6 snRNP stability might be a factor.

The broader consequences of these findings are that two RNA polymerases seem to cooperate to express proper levels of the RNA and protein components of the spliceosome. This is reminiscent of the observation that the production of the ribosome—a distinct RNA–protein machine that requires the activities of Pols I, II, and III must be coordinated as cells grow and divide. Indeed, recent evidence suggests that RNA polymerase I (Pol I) activity controls synthesis of ribosomal protein and *5S rRNA* genes by Pols II and III [64]. In another example, the human protein Maf1 negatively regulates Pols I, II, and III-dependent transcription [47,48]. Our study also suggests that polymerase cooperation is likely a factor when short hairpin RNAs (shRNAs) are expressed under the control of the *U6* promoter for the purpose of RNA interference. Interestingly, spliceosomal snRNA levels vary considerably with respect to one another. For example, U1 and U2 snRNAs are 5- to 10-fold more abundant than U4, U5, and U6 snRNAs in tissue culture cells [62]. Presumably this balance of spliceosomal components is optimal to support spliceosome assembly, splicing, and recycling of spliceosomal components. Undeniably, production of the U6 snRNP alone requires products of Pol II (e.g., La, SART3/p110, and Lsm proteins) as well as Pol III (U6 snRNA). The data presented here clearly demonstrate that Pol II activity controls all aspects of spliceosomal snRNP biogenesis.

## Materials and Methods

### Cell culture.

Hela and pemHeLa cells were grown in DMEM (Gibco) supplemented with 10% fetal bovine serum and 1% penicillin/streptomycin (Gibco). The pemHeLa cell line is a variant of standard HeLa cells; it contains the rat *pem* homeobox gene stably integrated [65] and grows to a high density favourable for ChIP. Standard HeLa cells were used for all experiments involving transfection.

### Chromatin immunoprecipitation and real time PCR.

A modification of the technique described by Kuo et al. was used [66]. Briefly, 10^8^ pemHeLa or HeLa cells were grown for 9 h ± 10 μg/ml α-amanitin before crosslinking with 1% formaldehyde (final concentration) added directly to the medium. Cells were washed with PBS and collected. Cell pellets were resuspended in 2 ml SDS lysis buffer (1% SDS, 10 mM EDTA, 50 mM Tris-HCl, pH 8.1) containing complete protease inhibitor cocktail (Roche) and incubated for 10 min on ice. Cell extracts were sonicated with a Branson sonifier W-450 D at 30% amplitude with 15 × 10 s bursts resulting in 200–500 bp chromatin fragments and then centrifuged for 10 min at 14,000 rpm. A 200 μl aliquot of the extract was diluted 1:10 in ChIP dilution buffer (0.01% SDS, 1.1% Triton X-100, 1.2 mM EDTA, 16.7 mM Tris-HCl, pH 8.1, 167 mM NaCl) containing protease inhibitors. The chromatin solution was pre-cleared at 4 °C with sepharose beads for 1 h before overnight incubation (4 °C) with either (1) 10 μg of 8WG16 (Neoclone), (2) 5 μg of 4H8 recognizing both Pol II0 and Pol IIA (Abcam), (3) 4 μl of rabbit polyclonal anti-RNA Pol III 1900 directed against the RPC 155 subunit of human Pol III (a gift from Robert J. White, [67]), (4) 4 μl of anti-acetyl-histone H4 (Upstate), or (5) 10 μg of non-immune IgG (Sigma) as control. Complexes were immunoprecipitated with GammaBind G sepharose beads (Pharmacia Biotech) for 1 h at 4 °C. The beads were washed rocking for 4 min in each of the following buffers: Low Salt Immune Complex Wash Buffer (0.1% SDS, 1% Triton X-100, 2 mM EDTA, 20 mM Tris-HCl, pH 8.1, 150 mM NaCl), High Salt Immune Complex Wash Buffer (0.1% SDS, 1% Triton X-100, 2 mM EDTA, 20 mM Tris-HCl, pH 8.1, 500 mM NaCl), LiCl Immune Complex Wash Buffer (0.25 M LiCl, 1% NP-40, 1% deoxycholic acid, 1 mM EDTA, 10 mM Tris-HCl, pH 8.1) and twice in TE. The immune complexes were eluted in 1 % SDS and 50 mM NaHCO_3_ and crosslinks were reversed for 6 h at 65 °C. Samples were digested with proteinase K for 1 h at 45 °C and the DNA extracted with the Qiagen PCR purification kit.

DNA templates retrieved by ChIP were analyzed by qPCR on a Stratagene MX3000, using the SYBR Green method (ABsolute QPCR SYBR Green Rox Mix, AB Gene). The reaction volume was 20 μl, with 4 μl DNA template and 90–900 nM of each primer according to individual optimization. Primer sets distinguishing between different regions of the genes were designed by using the Primer3 program (http://fokker.wi.mit.edu/primer3/input.htm) and are available upon request. They were blasted against the human genome to verify their specificity. Moreover, primer sets were evaluated by conventional and qPCR to ensure that only uniformly sized products were amplified.

The relative proportions of coimmunoprecipitated gene fragments were determined based on the threshold cycle (Ct) for each PCR product. Data sets were normalized according to 2 ^Ct(unspec. ab) − Ct(spec. ab)^. The fold difference over background obtained for gene regions was further normalized to the value obtained with a primer pair amplifying an intergenic region on chromosome 10 where no annotated genes could be found. For every gene fragment analyzed, each sample was quantified in duplicate and from ≥3 independent ChIPs. SEM was determined for each fold difference above nonimmune control and intergenic control region.

### Construction of maxigenes.

To generate the *U6–1* maxi TTTT construct, *U6–1* maxigene plasmid DNA [22] was restricted with EcoN1 at position 66 of the *U6* transcribed sequence. Complementary oligos 5′-GTCCCGGGTGTTTTT-3′ and 5′- CAAAAACACCCGGGA-3′ containing a SmaI restriction site followed by a Pol III termination sequence were inserted by ligation. For the *U6–1* maxi control construct, primers Mut1 (5′-CGTGTCATCCTTGCCACCCGGGACGCAG-3′) and Mut2 (5′-CTGCGTCCCGGGTGGCAAGGATGACACG-3′) were used to delete the five thymidines by PCR mutagenesis using the QuickChange protocol (Stratagene) as suggested by the manufacturer.

### Transfection, quantitative PCR, and primer extension.

For transfection experiments, HeLa cells were grown to 60%–80% confluency on a 10 cm dish before the medium was changed to growth medium containing either 50 nM methyl α-amanitin oleate (a more cell-permeable derivate of α-amanitin; Merck) or DMSO. 1 μg of each *U6* maxigene plasmid (a gift from Gary G. Kunkel [22]) was transfected with CaPO_4_ [68]. The medium was replaced by fresh medium containing 50 nM methyl α-amanitin oleate 16 h later and growth was continued for 4 h until total RNA was extracted with TRIZOL (Invitrogen) according to the manufacturer's recommendation. Total RNA was briefly exposed to RNase-free DNase I (Ambion) and reverse transcribed to cDNA using oligo (dT)_18_ primer or gene-specific primers and SuperScript III Kit (Invitrogen). cDNA from maxigene transfected cells was amplified by qPCR and relative expression levels were determined using the 2^ΔΔCt^ method according to the Stratagene MX3000 recommendations, using 28S rRNA as calibrator.

For primer extension analysis, HeLa cells were grown to 40% confluency before CaPO_4_ cotransfection with 8 μg *U6* maxigenes and 5 μg EGFP C3 (Clonetech). After 48 h, total RNA was isolated and analyzed with the Primer Extension System (Promega) and the following reverse primers: GFP primer (5′-AGCTTGCCGTAGGTGGC-3′), control primer (5′-TGCCACCCGGGACGCAG-3′), TTTTT primer (5′-GCAAAAACACCCGGGACG-3′), and maxi primer (5′-CGCTTCACGCCTCGAGGG-3′), resulting in extension products of 158, 79, 83, and 109 or 114 bp, respectively. Products were separated by electrophoresis on 10% polyacrylamide gels containing 7 M urea and exposed to PhosphorImager plates.

### Hybrid selection.

HeLa cells were grown to 60%–80% confluency before CaPO_4_ transfection with 1 μg *tRNA^Arg^* maxigene [69]. After16 h, medium was replaced by DMEM with or without 10 μg/ml α-amanitin for 3 h, followed by addition of 250 μCi [^32^P] orthophosphate (GE Healthcare) for 6 h before total RNA was extracted with Trizol. After 5 min denaturation at 70 °C, RNA was allowed to hybridize with biotinylated oligos complementary to 5S rRNA together with either U6, U2 [70], or tRNA^Arg^ for 30 min in 60% buffer D containing 5% glycerol [71]. The oligos used were complementary to positions 88–108 for 5S rRNA, 82–101 for U6 snRNA, 31–45 for U2 snRNA, or 56–76 for tRNA^Arg^. RNA/DNA hybrids were immunoprecipitated with streptavidin beads for 30 min followed by 5 washes in 60% buffer D. Immunopurified RNA was extracted with phenol/chloroform and separated by electrophoresis on a 10% polyacrylamide gel containing 7 M urea. Band intensities of RNAs synthesized in the presence of α-amanitin were measured using a PhosphorImager and normalized to 5S rRNA intensities to account for IP bead loss and loading differences.

## Supporting Information

Figure S1 Genomic Location of the Five Active *U6 snRNA* Genes The 107 nucleotide U6 snRNA sequence was aligned to the human genome with BLAT (BLASTlike alignment tool; UCSC genome informatics site (http://genome.ucsc.edu/, freeze March 2006, [23,24]) and images of 3 kb surrounding genomic region exported. The designations of the *U6* genes are according to [22].
(1.6 MB PDF)

Figure S2 Transcriptional Activity of the *U6–1* Surrounding Region (A) Transcriptional activity of the *U6–1* neighbouring gene *BC033162* was analyzed by reverse transcription, followed by PCR. Total RNA from HeLa cells was treated with DNaseI or RNaseA and transcripts were reverse-transcribed with oligo d(T) or gene-specific primers for *PGK1* and *BC033162*. The abundance *BC033162* mRNA was analyzed by 27–30 cycles of PCR with primers amplifying a single exon. (B) Northern Blot with total HeLa RNA with probes specific for U6 snRNA together with probes covering 782 bp of U6 upstream region on − strand (lane 1) and + strand (lane 2) as well as probes covering 923 bp of downstream regions of either − strand (lane 3) or + strand (lane 4) revealed that no transcript derives from the *U6–1* locus other than U6 snRNA. Lane 5, total RNA stained with ethidium bromide; lane 6, size markers. Asterisks indicate background hybridization to 28S and 18S rRNA.
(1.3 MB PDF)

Figure S3 Accumulation Profile of Pol II and Pol III at the *U6–1*, *U6–7*, and *U6–8* Promoters (A, B) Diagrams of the *U6–1* and *U6–7/U6–8* gene regions, with black lines specifying the PCR amplicons identified by the central nucleotide. Boxes represent the position of the DSE. Crosslinked pemHeLa extracts were used for ChIP with primers amplifying fragments distributed 1 kb upstream and downstream of each gene. Antibodies specific for Pol II (4H8 C, D; 8WG16 E, F) and Pol III were used. X-axis values in (C, E, G) are relative to the *U6–1* transcription start site at position +1, whereas transcription start sites in (D, F, H) are at +1,302 (*U6–7*) and +2,426 (*U6–8*), relative to an arbitrary point upstream of *U6–7*. Data points are placed according to the center positions of the PCR products along the region. The peak value for each data set was set to 100% and all data points normalized accordingly. Error bars represent the SEM of at least three independent experiments.
(84 KB PDF)

Figure S4 Transcriptionally Active Pol II Large Subunit Accumulates at the *U6–1* Promoter Crosslinked pemHeLa extracts were used for ChIP with Pol II CTD Ser5 Antibody H14, with primers amplifying fragments distributed 1kb upstream and downstream of U6–1 snRNA gene region. All values are relative to nonimmune IgG and normalized to an intergenic control region. Error bars represent the standard deviation of two independent experiments.
(42 KB PDF)

### Accession Numbers

Accession numbers for genes mentioned in this paper from the National Center for Biotechnology Information database (http://www.ncbi.nlm.nih.gov) are tRNALeu (X04117), tRNATyr (M55612), LDHA (NM_005566), PGK1 (NM_000291), 5S rRNA (X51545), 28S rRNA (NR_003287), U2 snRNA (X59360), *U1 snRNA* (J00318), *U6 snRNA* (X07425).

## Abbreviations

ChIP: chromatin immunoprecipitation
CTD: C-terminal domain
DSE: distal sequence element
GFP: green fluorescent protein
PGK1: phosphoglycerate kinase
Pol I: RNA polymerase I
Pol II: RNA polymerase II
Pol III: RNA polymerase III
PSE: proximal sequence element
qPCR: quantitative PCR
SEM: standard error of the mean
snRNA: small nuclear RNA
snRNP: small nuclear ribonucleoprotein particle
