# Supplementary material for: Extragenic Accumulation of RNA Polymerase II Enhances Transcription by RNA Polymerase III
Source: PLoS Genet. 2007 Nov 23;3(11):e212. doi: 10.1371/journal.pgen.0030212 (PMC2082468; doi:10.1371/journal.pgen.0030212)
Supplement: Supplementary file 1 [file pgen.0030212.sg001.pdf]

# U6-1

minus strand
65919331 bp -
65919437 bp

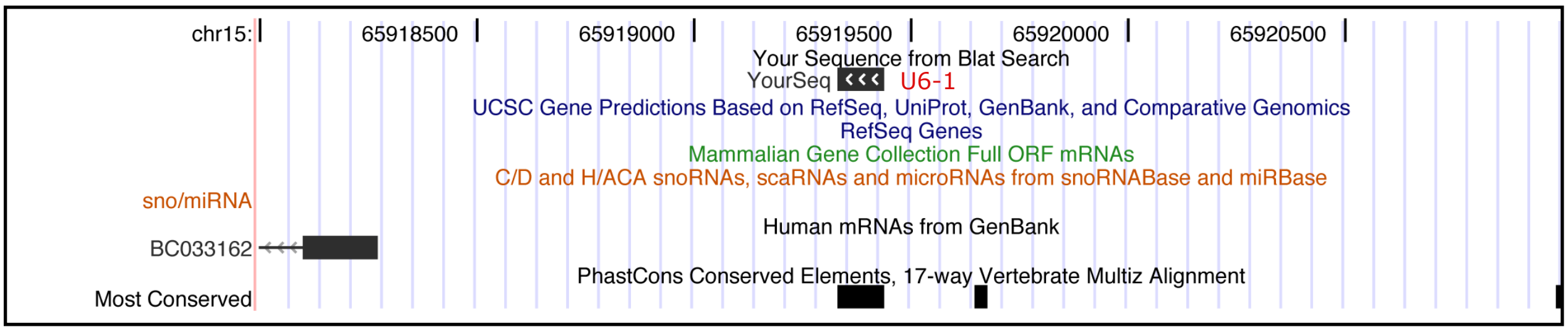


# U6-2

plus strand
972521 bp -
972627 bp

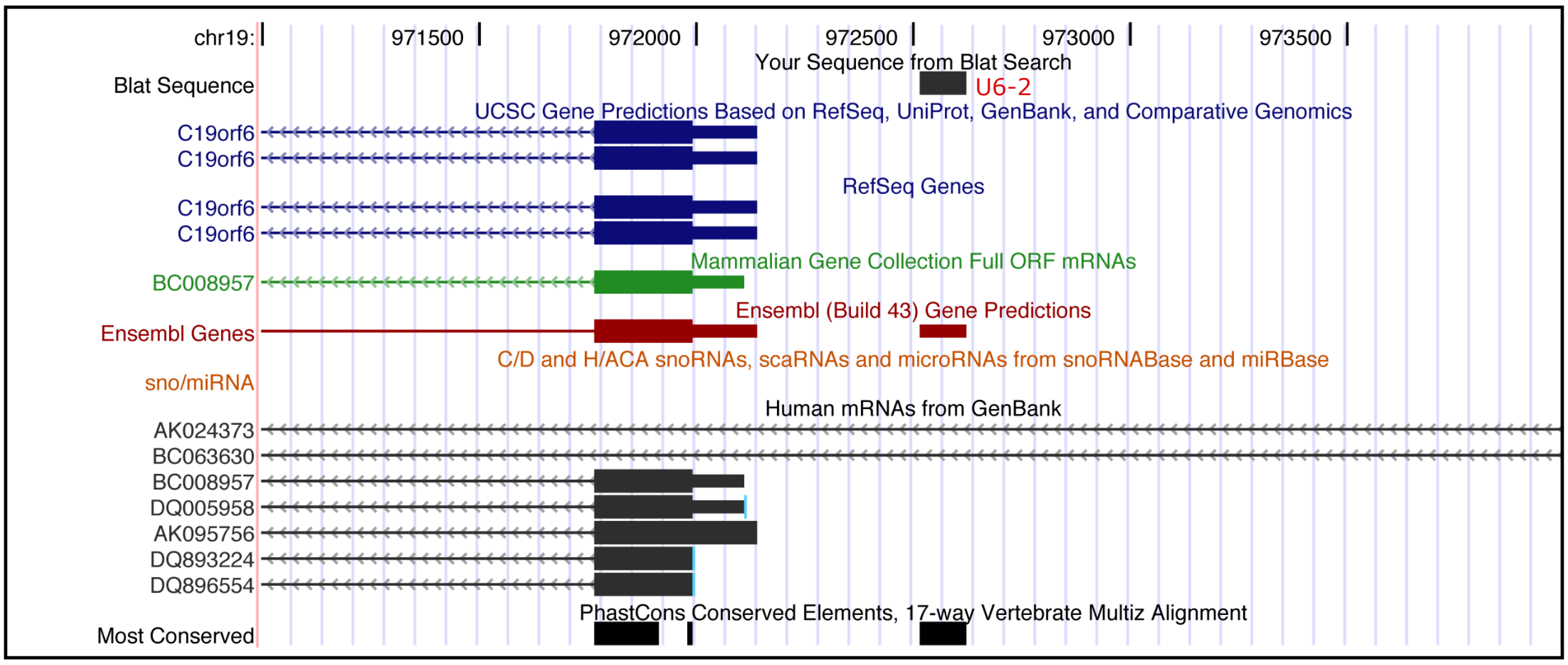


# U6-7

plus strand
31741002 bp -
31741107 bp

# U6-8

minus strand
31742121 bp -
31742226 bp

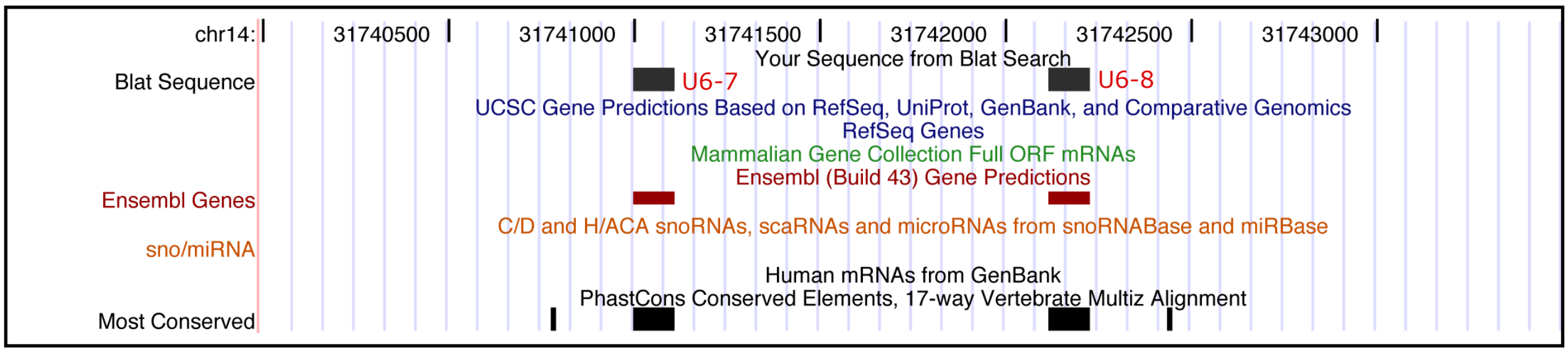


# U6-9

plus strand
844484 bp -
844590 bp

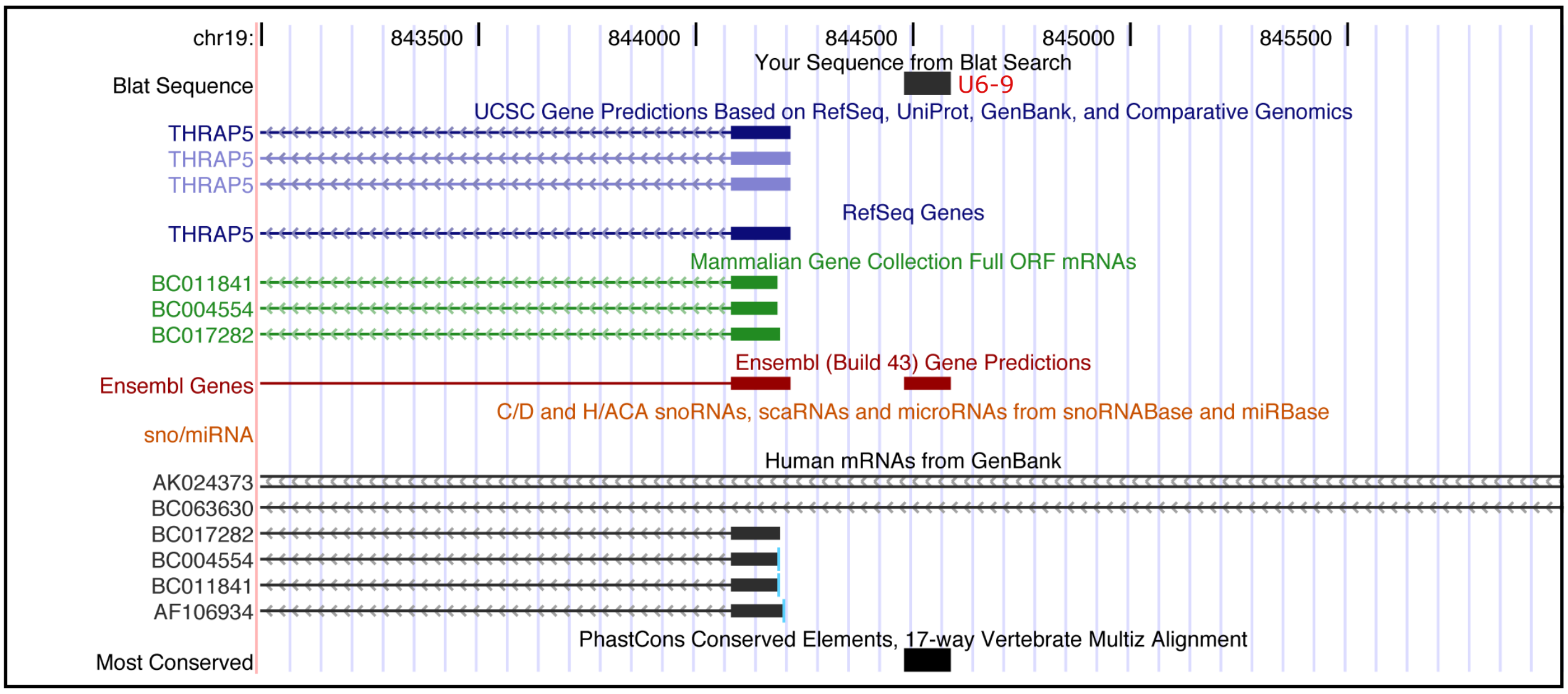


Supplementary Figure 1
