# Supplementary material for: Extragenic Accumulation of RNA Polymerase II Enhances Transcription by RNA Polymerase III
Source: PLoS Genet. 2007 Nov 23;3(11):e212. doi: 10.1371/journal.pgen.0030212 (PMC2082468; doi:10.1371/journal.pgen.0030212)
Supplement: Supplementary file 3 [file pgen.0030212.sg003.pdf]

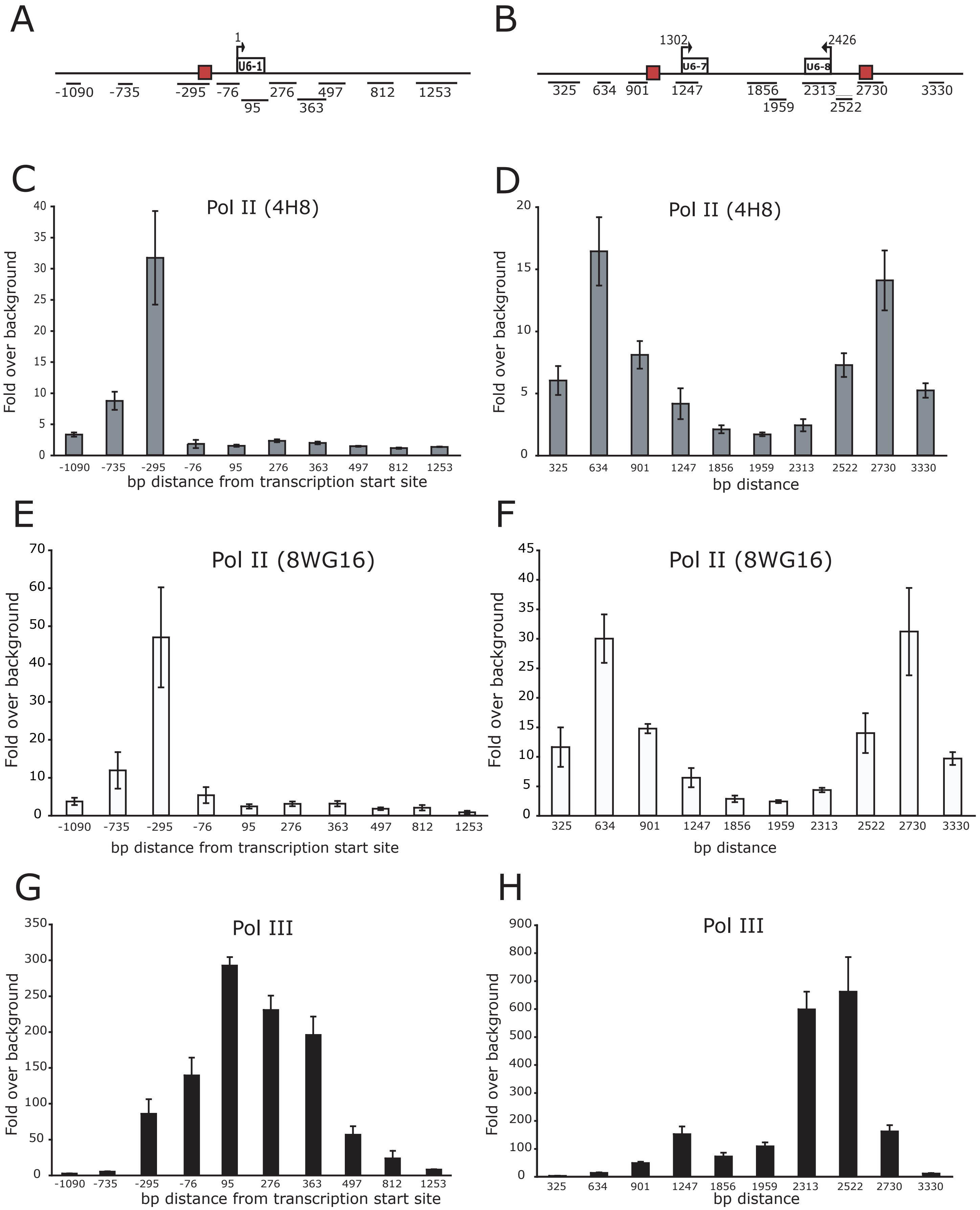

Supplementary Figure 3
